# Mitochondrial Iron Metabolism as a Potential Key Mediator of PD-L1 Thermal Regulation

**DOI:** 10.3390/cancers16223736

**Published:** 2024-11-05

**Authors:** Gizzy Keeler, Stephenson B. Owusu, Mario Zanaty, Michael S. Petronek

**Affiliations:** 1 Department of Radiation Oncology, University of Iowa, Iowa City, IA 52242-1181, USA; gizzy-keeler@uiowa.edu (G.K.);; 2 Department of Neurosurgery, University of Iowa, Iowa City, IA 52242-1181, USA

**Keywords:** laser interstitial thermal therapy, glioblastoma, immunotherapy, PD-L1, mitochondrial iron metabolism

## Abstract

Glioblastoma (GBM) is the most common primary brain malignancy in the U.S. with a 5-year overall survival < 5% despite an aggressive standard of care. Laser interstitial thermal therapy (LITT) is a surgical approach to treating GBM that has gained traction, providing a safe option for reducing intracranial tumor burden. LITT is believed to potentially modulate GBM immune responses; however, the biochemical mechanisms underlying the modulation of immune checkpoints in GBM cells have been poorly characterized. The present study aimed to preliminarily evaluate the effects of thermal therapy and radiation on PD-L1 modulation in vitro, as a function of IDH mutational status. U87 cells and their IDH-mutant counterpart (U87^R132H^), which was generated using a crispr-cas9 knock-in approach, were utilized for this preliminary evaluation. Cell heating was achieved by harvesting with trypsin centrifugation where the cell pellets were treated on a heat block for the associated time and temperature. Following thermal therapy, cells were resuspended and irradiated using a 37-Cesium irradiator at 0.6 Gy min^−1^. Immediately following treatment, cells were either plated as single cells to allow colonies to form, and stained with Coomassie blue to be counted approximately 10–14 days later or harvested for Western blot analysis. Cell lysates were analyzed for PD-L1 expression with respect to various iron metabolic parameters (mortalin (HSPA9), transferrin receptor, and ferritin heavy chain) using a Western blotting approach. In both U87 and U87^R132H^ cell lines, thermal therapy showed a temperature-dependent cell-killing effect, but U87^R132H^ cells appeared more sensitive to thermal treatment when treated at 43 °C for 10 min. Moreover, thermal therapy had minimal effects on cell responses to 2 Gy irradiation. Treatment with thermal therapy downregulated PD-L1 expression in U87^R132H^ cells, which was associated with increased expression of the mitochondrial iron metabolic enzyme, HSPA9. Thermal therapy reversed the radiation-induced overexpression of PD-L1, transferrin receptor, and ferritin heavy chain in U87^R132H^ cells. No effects were observed in wild-type U87 cells. Moreover, Ga(NO_3_)_3_ depleted mitochondrial iron content which, in turn, significantly enhanced the sensitivity of U87^R132H^ cells to thermal therapy and 2 Gy irradiation and caused a significant increase in PD-L1 expression. These results suggest that thermal therapy alone can modulate the immune checkpoint PD-L1. This effect was more pronounced when thermal therapy was combined with radiation. Mechanistically, mitochondrial iron trafficking through HSPA9 may coordinate the regulation of PD-L1 in the context of thermal therapy and ionizing radiation, which can be targeted with gallium-based therapy. These novel, preliminary findings warrant further mechanistic investigations in pre-clinical models of LITT.

## Simple Summary

Laser interstitial thermal therapy (LITT) is a novel surgical therapy that is currently being investigated for the management of glioblastoma tumors. There are some reports that suggest that the use of LITT is capable of modulating glioblastoma immune responses, however, mechanistic understanding at the cellular level is limited. In this study, we evaluate the effects of LITT relevant thermal dosing (43 °C, 10 min) on PD-L1 expression in IDH mutant and wild-type U87 cells. Here we observed that thermal therapy can reverse radiation-induced PD-L1 expression which is associated with the induction of mortalin—a heat shock protein that is central to iron-sulfur cluster biogenesis in the mitochondria. Moreover, treatment with gallium which inhibits mitochondrial iron uptake reverses this effect and enhances sensitivity to both radiation and thermal therapy. Thus, mitochondrial iron metabolism may serve as a critical biochemical regulator of glioblastoma immune checkpoint signaling.

## 1. Introduction

Glioblastoma (GBM) is the most common primary brain malignancy (≈14,000 diagnosed cases per year) in the United States [1,2,3]. The median survival is only 14–16 months and 5-year overall survival (OS) is under 5% despite aggressive standard of care (SOC) treatments including ionizing radiation and temozolomide [4,5]. In addition to the poor outcomes seen for GBM patients in general, in some circumstances of deep-seated tumor growth, surgical resection is not viable. For inoperable GBM, where only a diagnostic biopsy can be performed, the median overall survival is only 9.4 months [6]. For these patients, current non-operative treatments are less effective and lead to even worse outcomes [7].

Laser interstitial thermal therapy (LITT) is a modernized intraoperative approach to promote thermal ablation and has been shown to potentially enhance patient responses in those with inoperable tumors or for those who have exhausted therapeutic options [8,9]. LITT works by heating the tumor bed to 43 °C for 10 min. This therapy provides a safe option for reducing intracranial tumor burden for patients who are poor surgical candidates but does not significantly increase progression-free survival [7]. To date, there have been several clinical trials investigating LITT in both primary and recurrent GBM patients (e.g., NCT05318612, NCT05663125, NCT05296122) with ongoing studies interrogating the effectiveness of LITT in combination with ionizing radiation (NCT04181684, NCT04699773). Within the neurosurgical community there may be interest in LITT usage as it has been associated with the preservation of cognitive function and stabilization of KPS scores [10]. However, LITT does still pose a risk of potential adverse events including seizures, hemorrhage, and worsening edema with a particular risk for patients with tumors located near or contained within sensitive anatomical regions [11,12]. From a surgical neuro-oncology perspective, the patient responses also help to maintain attractiveness of LITT as a therapeutic strategy. An exploratory study of 91 subjects receiving 100 LITT treatments at a single institution revealed that 61% of patients were alive with 72% local control at 7.2-month follow-up with extent-of-ablation > 85% resulting in a significant increase in the time to recurrence [12]. A study investigating 29 new and 60 recurrent patients with unresectable GBM (IDH wild-type) who were treated with LITT at 14 participating U.S. institutions revealed a median overall survival of 9.73 months for newly diagnosed and 8.97 months for recurrent patients with minimal adverse events [8]. However, the median overall survival for patients receiving radiation and temozolomide post-LITT was 16.14 months with MGMT promoter methylation, adjuvant chemotherapy within 12 weeks, and tumor volume (<3 cc) being associated with increased overall survival. Another retrospective study of LITT of 69 patients (including those with newly diagnosed and recurrent GBM) revealed that the overall survival of the total cohort was 12 months from the time of LITT [9]. In this study, adjuvant chemotherapy was also associated with prolonged survival; however, neither diagnosis stage (primary versus recurrence) nor IDH mutational status was predictive of survival. Taken together, LITT appears to be a viable clinical option in combination with radiation and chemotherapy for GBM patients.

Currently, the biochemical effects of LITT on GBM cells is still unclear. Mechanistically, hyperthermal ablation (41–45 °C) has the potential to generate enhanced reactive oxygen species to promote DNA damage through either direct DNA breaks or impaired DNA replication [13,14]. Such DNA damage that occurs due to hyperthermal ablation may alter gene expression of immune markers in tumor cells, such as programmed death ligand–1 (PD-L1), a critical biomarker for immune checkpoint inhibitor (ICI) therapy [15]. Recent literature surrounding LITT suggests that its use addresses some important limitations of ICIs. LITT has been shown to disrupt the peritumoral BBB, increasing permeability and potential for delivery of therapeutic agents [16]. This disruption appears to occur both through transcellular transport mechanisms as well as by physical disruption of tight junctions [17]. In addition, the thermocoagulation of tumor tissue by LITT prompts the TME to turn from immunologically silent to active, possibly due to production of HSP and excretion of exosomes by tumor cells, enhancing the immune-regulated cytotoxic killing of tumor cells [18]. Finally, a recent study using thermotherapy, induced locally via iron oxide nanoparticles (i.e., magnetic hyperthermia), demonstrated a strong increase in PD-L1 expression when heated to 55.5 °C [19]. This upregulation of PD-L1 provides a clear target for the use of ICIs (e.g., anti-PD-1, pembrolizumab) in combination with LITT. Thus, it appears that although LITT alone may not significantly increase clinical outcomes, it may be a viable option in combination with ICIs due to its ability to disrupt the peritumoral BBB and potentiate anti-tumor immune responses. A few clinical trials have tested this model, with mixed results surrounding prolonged responses to immunotherapy and safety of LITT + immunotherapy [6,20,21]. With the growing interest in using LITT as treatment strategy in GBM, particularly with respect to ICI therapy, the goal of this study was to preliminarily evaluate the effects of clinically relevant thermal doses on PD-L1 expression in isocitrate dehydrogenase wild-type and mutant U87 model systems to help contextualize the potential immune modulatory effects of thermal therapy.

## 2. Materials and Methods

### 2.1. Cell Culture

All glioma cells (U87, ATCC HTB-14, U87^R132H^, and ATCC HTB-14IG) were cultured in DMEM-F12 media (15% FBS, 1% penicillin-strep, 1% Na-pyruvate, 1.5% HEPES, 0.1% insulin, and 0.02% fibroblast growth factor) and grown to 70–80% confluence at 21% O_2_ before experimentation. U87^R132H^ cells were generated at ATCC using a c.395G>A knock-in mutation encoding IDH1-R132H protein expression. Both cell lines were validated and authenticated before use. Cells were grown at 37 °C with 21% O_2_.

### 2.2. In Vitro Cell Treatments

To model thermal ablation using an in vitro model system, untreated, exponentially growing cells were grown to 70–80% confluence, trypsinized to detach the cells, and centrifuged at 1200 rpm for 5 min to collect cell pellets. Cell pellets were then placed in a temperature-controlled head block set to 43, 46, or 50 °C for 3 or 10 min. Cell pellets were either resuspended in standard cell culture media to be plated for colony formation analysis to assess toxicity, or harvested for Western blot analysis immediately following heating. For radiation treatments, cell pellets were resuspended in cell culture media and irradiated with 2 Gy using a ^37^Cs source immediately prior to plating. Cell pellets were harvested in cold PBS for Western blot analysis.

### 2.3. Colony Formation

Immediately following the thermal ablation of the cell pellet, cells were resuspended and plated as single cells (500–750 cells). Cells were allowed up to 10 days to form colonies (≥50 cells) before being stained. For analysis, cells were fixed with 70% ethanol, stained with Coomassie blue, and the number of colonies was counted manually under a microscope. The plating efficiency for each group was calculated using the following formula:

$$Plating\;efficiency\left(\%\right)=\left(\frac{\#\;colonies\;counted}{\#\;cells\;plated}\right)*100$$

The plating efficiency from each group was normalized to their respective control to generate a normalized survival fraction.

### 2.4. Western Blotting

Cells were lysed in RIPA buffer (Sigma Aldrich, St. Louis, MO, USA), and total protein of the supernatant was quantified using a DC™ protein assay kit (Bio-Rad, Hercules, CA, USA).

SDS-PAGE was carried out using 4–20% precast polyacrylamide gel (Bio-Rad, Hercules, CA, USA). The resolved proteins were electro-transferred to 0.22 um pore size PVDF membranes (Bio-Rad, Hercules, CA, USA) at room temperature for 10 min using the Trans-Blot Turbo System (Bio-Rad, Hercules, CA, USA). The PVDF membranes were blocked in a 5% milk solution for 1 h on an orbital shaker at room temperature. Membranes were then incubated overnight with specific primary monoclonal or polyclonal antibodies: PD-L1/CD274 monoclonal antibody (1:1000 dilution; Proteintech, Rosemont, Il, USA), transferrin receptor (ProteinTech), ferritin heavy chain polyclonal antibody (1:1000 dilution; ProteinTech), GRP75 (mortalin, HSPA9), and polyclonal antibody (1: 2500, ProteinTech) ProteinTech). The membranes were washed 3×, 5 min each with 1× TBST (Bio-Rad) and were incubated with either goat anti-mouse (1:5000; Cell Signaling, Danvers, MA, USA) or anti-rabbit (1:5000; Cell Signaling) conjugated with HRP for 1 h at room temperature. The membranes were then washed with 1× TBS-T and the signals were developed with a chemiluminescent kit (Super Signal West Pico & Super Signal West Femto, Thermo Scientific, Tempe, AZ, USA) and exposed on an X-ray film (Research Products International, Mount Prospect, IL, USA).

## 3. Results

**Figure 1:**
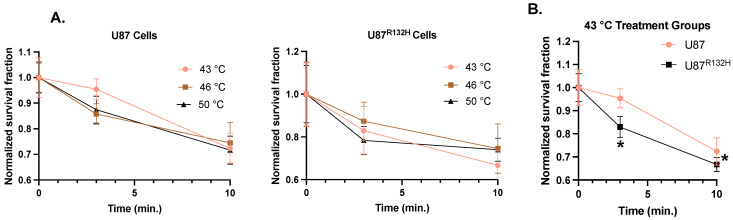
**Temperature-dependent cell killing of IDH-mutant and wild-type glioma cells.** (**A**) U87 and U87^R132H^ cells were heated to 43, 46, and 50 °C for 3 and 10 min prior to being plated for clonogenic survival (left and center panels, respectively). (**B**) Relative sensitivity of U87 and U87^R132H^ cells when heated to 43 °C for 10 min. Error bars represent mean ± SD (*n* = 6 per group) with * *p* < 0.05 using a two-way ANOVA test.

To first establish this in vitro model system, clonogenic survival analysis was conducted to evaluate the cytotoxic effects of thermal therapy on U87 and U87^R132H^ cells. In both cell lines, time-dependent cell killing was observed (Figure 1A). However, there was not a notable temperature dependence in either cell line. The standard thermal dose threshold for LITT is 43 °C for 10 min, therefore, a dose–response assessment was performed with cells being treated for 3 and 10 min at 43 °C to compare the relative sensitivity of the two cell lines [22]. Significant cell killing was observed at 43 °C for 10 min in both cell lines (Figure 1B). However, significant sensitivity was only observed in U87^R132H^ cells when treated with 43 °C for 3 min. These findings suggest that there was increased sensitivity to mild thermal therapy in IDH-mutant U87 cells.

**Figure 2:**
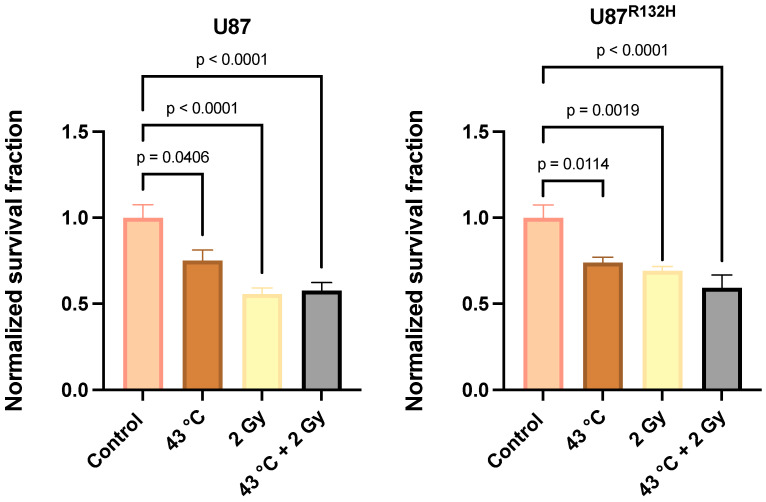
**Effects of thermal therapy on glioma cell radiosensitivity.** U87 and U87^R132H^ cells were heated to 43 °C for 10 min prior to being treated with 2 Gy irradiation and plated to assess colony formation. Error bars represent mean ± SD (n = 6 per group) with *p*-values generated using a one-way ANOVA test.

Ionizing radiation is a standard therapy for the management of GBM [4]; thus, the next goal of this study was to investigate the effects of thermal therapy on cellular radiosensitivity. Moreover, hyperthermia therapy is widely regarded as a potent radiosensitizing approach [23]. However, a robust enhancement of radiation sensitivity (2 Gy) was not observed in either U87 or U87^R132H^ cells (Figure 2). Meanwhile, it can be noted that although minimal, any potential additive effects only seemed to appear in the U87^R132H^ cells. Therefore, the use of thermal therapy in combination with radiation may require further mechanistic considerations to effectively modulate sensitivity.

**Figure 3:**
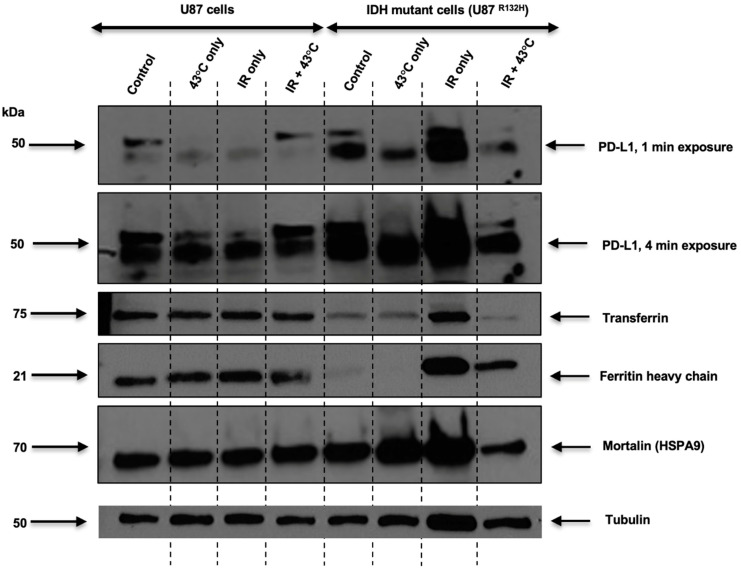
**Thermal reversal of radiation-induced PD-L1 expression is associated with induction of mortalin.** Western blot analysis of PD-L1, TfR, Ft-H, and mortalin expression in U87 and U87^R132H^ cells that had been heated to 43 °C for 10 min prior to being treated with 2 Gy irradiation. Uncropped western blots are provided in supplementary materials.

One of the main goals of this study was to assess the effects of thermal therapy on PD-L1 expression as a potential immune modulatory effect. Consistent with the mild effects of thermal therapy and radiation, only U87^R132H^ cells revealed an observable effect of thermal therapy or radiation on PD-L1 expression. In U87^R132H^ cells, 2 Gy radiation enhanced PD-L1 expression, which was effectively reversed by a 10 min, 43 °C treatment (Figure 3). To provide potential mechanistic insights into this compensatory effect, mortalin (HSPA9) was interrogated as it a heat-shock protein that has been shown to directly inhibit PD-L1 expression in glioblastoma cells [24]. Consistently, mortalin expression was elevated by both thermal therapy and radiation which suggests that the thermal enhancement of mortalin may be central to the reversal of radiation-induced PD-L1. Because mortalin serves as a mitochondrially-localized, iron–sulfur cluster chaperone protein, the central iron metabolic proteins transferrin receptor (TfR) and ferritin heavy chain (FtH) were interrogated to determine if this effect was associated with iron metabolism [25]. In line with the PD-L1 changes, radiation enhanced both TfR and FtH expression which was reversed with combined therapy. Taken together, these results showcase that thermal therapy and radiation can modulate tumor immune signaling and suggest that this effect is related to iron metabolism, corroborated through mortalin in the mitochondria.

**Figure 4:**
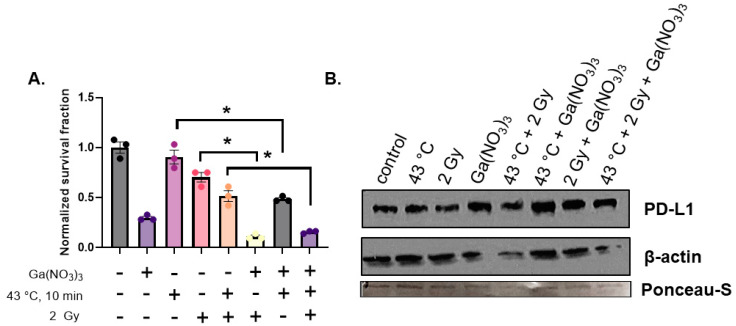
**Gallium enhances the toxicity of thermal therapy and ionizing radiation.** (**A**) Clonogenic survival analysis of U87^R132H^ cells treated with ± 500 µM Ga(NO_3_)_3_ for 24 h prior to collection and treatment at ± 43 °C for 10 min followed by 2 Gy irradiation. Error bars represent mean ± SEM (*n* = 3) where * *p* < 0.05 using a one-way ANOVA test with a post-hoc Tukey’s analysis for multiple comparisons. (**B**) Western blot analysis of PD-L1 cells treated with ± 500 µM Ga(NO_3_)_3_ for 24 h prior to collection and treatment ± 43 °C for 10 min, followed by 2 Gy irradiation. β-actin was used as a loading control, but due to variability in β-actin, the membrane was stained with Ponseau-S as an additional loading control (band shown for 37 kDa).

Because mortalin exists as part of the iron–sulfur cluster biogenesis machinery in the mitochondria [26], it was hypothesized that thermal modulation of PD-L1 is due to altered mitochondrial iron trafficking. Moreover, iron–sulfur cluster formation is intimately connected to cell survival, as many critical DNA metabolic enzymes contain [4Fe-4S]^2+^ clusters; therefore, it can also be hypothesized that the enhanced mortalin expression is associated with cell survival and can be targeted therapeutically [27]. To test this hypothesis, cells were treated with 500 µM Ga(NO_3_)_3_ for 24 h, which had be shown to cause a decrease in mitochondrial iron content [28]. Consistent with the hypothesis, the addition of Ga(NO_3_)_3_ significantly enhanced the cytotoxicity of thermal therapy and radiation (Figure 4A). Moreover, Ga(NO_3_)_3_ reversed the effects of thermal therapy and radiation on PD-L1 expression (Figure 4B). These results further support the hypothesis that mitochondrial iron metabolism is a critical target for both cellular radiation responses and tumor-immune signaling.

## 4. Discussion

As interest in the clinical utility of LITT continues to increase, so does the demand for more robust mechanistic investigations to better contextualize and optimize its utility. There is currently a considerable interest within the neuro-oncologic research community to better contextualize the use of immunotherapy in GBM therapy [29,30]. A recent case report of a 51-year-old male with a recurrent GBM, who underwent LITT therapy and a subsequent tumor biopsy, revealed that LITT caused a significant increase in activated CD8 T cells, activated macrophages, and PD-L1 expression [31]. The robust increase in tumor immune infiltrates was associated with long-term survival as the patient was alive 9 years after diagnosis and serial, post-operative imaging over 3 years revealed no evidence of tumor recurrence. Recently, clinical trials have been developed to investigate the usefulness of immunotherapy in combination with LITT for patients with recurrent GBM (NCT03277638, NCT03341806). Thus, there is considerable interest in exploring the immune modulatory effects of LITT.

Thus, the overarching goal of this study was to preliminarily investigate the effects of thermal therapy on PD-L1 expression in IDH wild-type and mutant glioma cells. We found that thermal therapy reduced PD-L1 expression and reversed the radio-induction of PD-L1 in IDH-mutant cells. In this study, it has been observed that, in vitro, there is minimal thermal modulation of PD-L1 expression in IDH wild-type cells. However, these preliminary studies were conducted in U87 cells alone and should be further corroborated using patient-derived cells to determine if this truly is an IDH-dependent effect and should be interpreted as such with caution.

**Figure 5:**
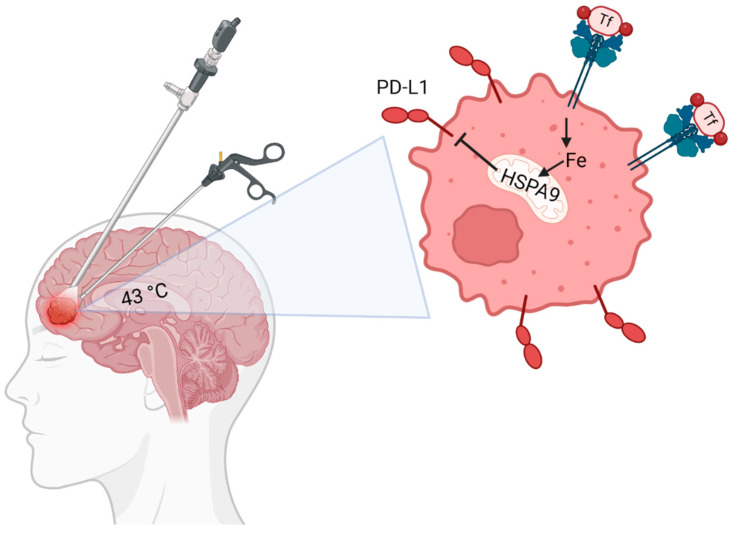
**Working biochemical model.** These results have led to the following working model: It can be hypothesized that LITT-induced mortalin (HSPA9) may alter Fe-S biogenesis rates to downregulate both TfR expression and PD-L1 expression. This working hypothesis requires further investigation to evaluate the causal relationship(s) between mortalin, TfR, and PD-L1 in the context of thermal therapy.

What may be most intriguing about these results lies in the potential for a new therapeutic target in mortalin (HSPA9) by corroborating PD-L1 and cell survival through mitochondrial iron metabolism (Figure 5). Our observation that heat-induced mortalin may be counteracting the radio-induction of PD-L1 is consistent with a previous study revealing that mortalin is a direct, negative regulator of PD-L1 [24]. Mechanistically, mortalin is a mitochondrial protein that serves as a key iron–sulfur cluster chaperone, connects the early/late acting biosynthesis machinery, and is required for the completion of functional iron–sulfur-cluster enzymes [26]. Consistent with thermal enhancement of mortalin and its potential connection to iron metabolic regulation is the downregulation of TfR and FtH. Both TfR and FtH are central iron metabolic enzymes whose expression is tightly regulated at the mRNA level in an iron–sulfur-cluster-dependent manner, thus, alterations in iron–sulfur-cluster formation would likely be reflected in TfR and FtH expression [32,33]. Importantly, thermal therapy completely reversed the enhancement of both PD-L1 and TfR associated with ionizing radiation. The associations of ionizing radiation and TfR are currently unknown; however, it has been shown that enhanced TfR expression is associated with worse patient outcomes, which suggests that thermal therapy in combination with radiation may impair tumor proliferation and progression [34,35]. Therapeutically, Ga(NO_3_)_3_, which has been shown to deplete mitochondrial iron, reversed the thermal regulation of PD-L1 and significantly enhanced the cell-killing effects of both thermal therapy and radiation [28]. It was difficult to distinguish if Ga(NO_3_)_3_ in combination with LITT and radiation was more toxic than Ga(NO_3_)_3_ and radiation alone due to the extreme toxicity from Ga(NO_3_)_3_ and radiation. Moreover, the use of a U87 model system serves as a useful proof of concept; however, these cells are not reflective of real GBM tumors, particularly considering that the U87^R132H^ represents an idealized, single mutation, which is a limitation of this study. Thus, it is unclear if this effect is specific to IDH-mutant tumors and requires more robust interrogation. These types of experiments should ideally be conducted in patient-derived glioma cells that intrinsically harbor IDH mutations in comparison to those that are IDH wild-type, as this would be more representative of a real-world scenario and account for greater genetic variability beyond the idealized U87^R132H^ model. Overall, these intriguing preliminary results suggest the novel hypothesis that mitochondrial iron trafficking is an important regulatory feature of PD-L1 expression that can be exploited therapeutically using gallium-based therapies to enhance the efficacy of LITT, radiation, and/or immune checkpoint inhibitors.

## 5. Conclusions

With the current interest in utilizing LITT for GBM management, particularly with respect to tumor immune responses, a more robust understanding of the biochemical mechanisms driving these effects is imperative. In this study, it has been observed that thermal therapy can reverse radiation-induced PD-L1 expression. This effect is associated with the induction of mortalin expression, a key intermediate of iron-sulfur cluster biogenesis in the mitochondria. Importantly, gallium is capable of reversing this effect, underscoring the potential importance of mitochondrial iron metabolism in regulating tumor immune checkpoints and suggesting that gallium may serve as a therapeutic option to enhance LITT and radiation therapy in GBM.

## Data Availability

The original contributions presented in the study are included in the article, further inquiries can be directed to the corresponding authors.

## Supplementary Materials

The following supporting information can be downloaded at: https://www.mdpi.com/article/10.3390/cancers16223736/s1, Uncropped western blots.
