# Supplementary figures and images for: Mitochondrial Iron Metabolism as a Potential Key Mediator of PD-L1 Thermal Regulation
Source: Cancers (Basel). 2024 Nov 5;16(22):3736. doi: 10.3390/cancers16223736 (PMC11592209; doi:10.3390/cancers16223736)

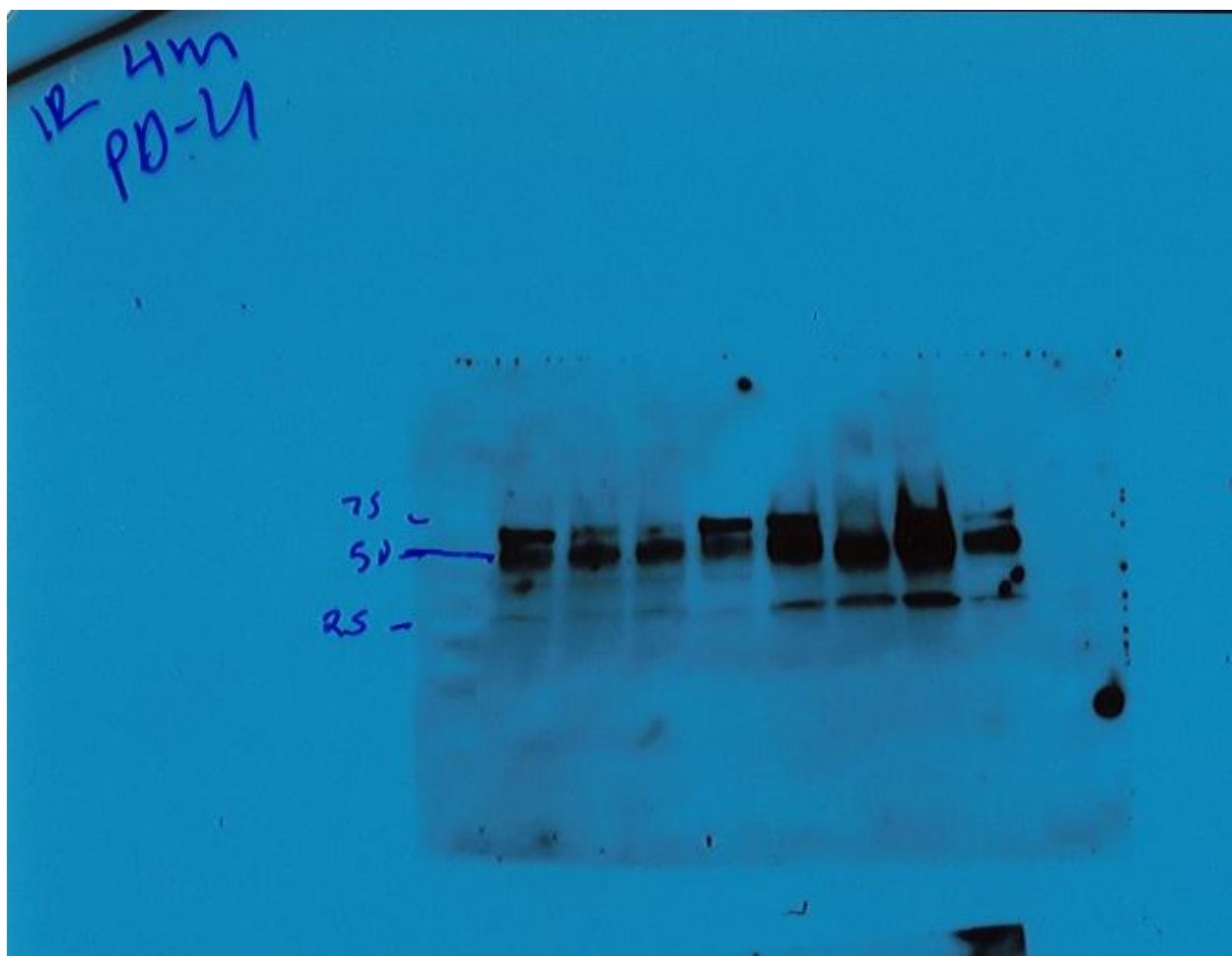

5m  
12 exp  
Transf.

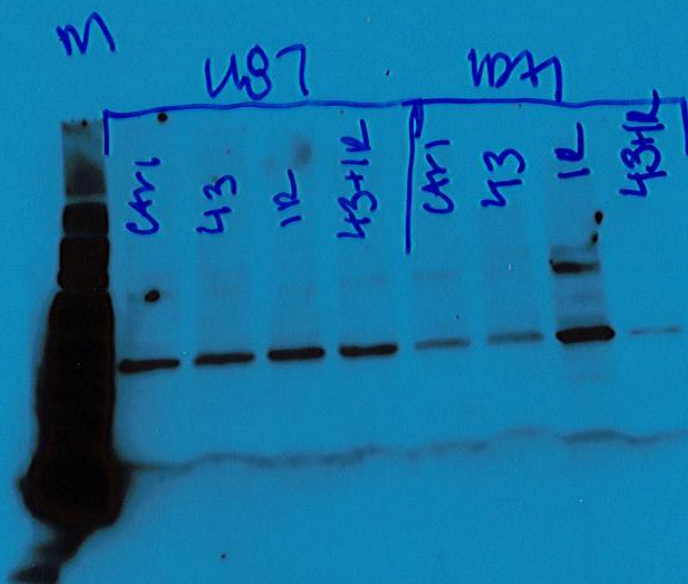

FtH G1337  
8.5.24

30s.

Exp R1

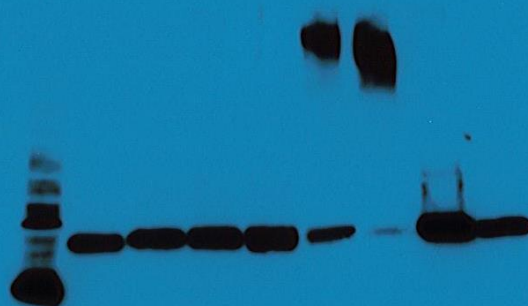

- FtH

305

12  
GAP + HSP

U87      IDH cells  
 12  
 C 43 12 43    C 43 12 43

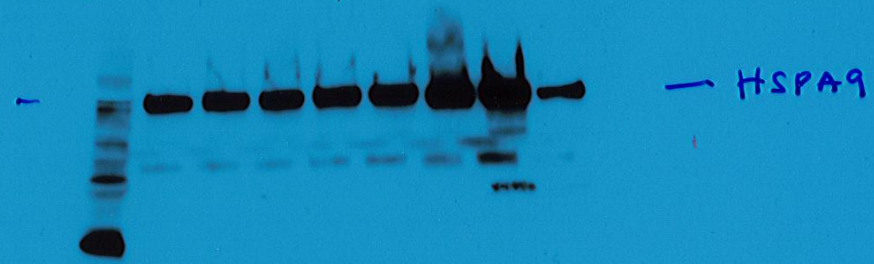

U87      IDH

50 -

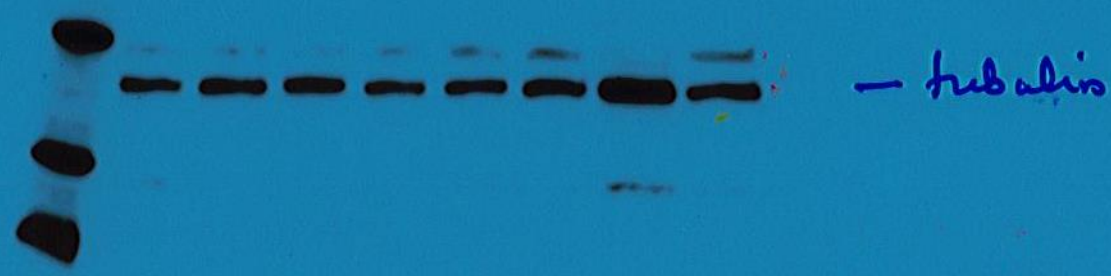

Supplement: Supplementary file 1 [file cancers-16-03736-s001.zip › cancers-3264951-supplementary.pdf]
